# *Vital Signs*: Trends in Incidence of Cancers Associated with Overweight and Obesity — United States, 2005–2014

**DOI:** 10.15585/mmwr.mm6639e1

**Published:** 2017-10-06

**Authors:** C. Brooke Steele, Cheryll C. Thomas, S. Jane Henley, Greta M. Massetti, Deborah A. Galuska, Tanya Agurs-Collins, Mary Puckett, Lisa C. Richardson

**Affiliations:** ^1^Division of Cancer Prevention and Control, CDC; ^2^Division of Nutrition, Physical Activity, and Obesity, CDC; ^3^Division of Cancer Control and Population Sciences, National Cancer Institute, Rockville, Maryland.

## Abstract

**Background:**

Overweight and obesity are associated with increased risk of at least 13 different types of cancer.

**Methods:**

Data from the United States Cancer Statistics for 2014 were used to assess incidence rates, and data from 2005 to 2014 were used to assess trends for cancers associated with overweight and obesity (adenocarcinoma of the esophagus; cancers of the breast [in postmenopausal women], colon and rectum, endometrium, gallbladder, gastric cardia, kidney, liver, ovary, pancreas, and thyroid; meningioma; and multiple myeloma) by sex, age, race/ethnicity, state, geographic region, and cancer site. Because screening for colorectal cancer can reduce colorectal cancer incidence through detection of precancerous polyps before they become cancerous, trends with and without colorectal cancer were analyzed.

**Results:**

In 2014, approximately 631,000 persons in the United States received a diagnosis of a cancer associated with overweight and obesity, representing 40% of all cancers diagnosed. Overweight- and obesity-related cancer incidence rates were higher among older persons (ages ≥50 years) than younger persons; higher among females than males; and higher among non-Hispanic black and non-Hispanic white adults compared with other groups. Incidence rates for overweight- and obesity-related cancers during 2005–2014 varied by age, cancer site, and state. Excluding colorectal cancer, incidence rates increased significantly among persons aged 20–74 years; decreased among those aged ≥75 years; increased in 32 states; and were stable in 16 states and the District of Columbia.

**Conclusions:**

The burden of overweight- and obesity-related cancer is high in the United States. Incidence rates of overweight- and obesity-related cancers except colorectal cancer have increased in some age groups and states.

**Implications for Public Health Practice:**

The burden of overweight- and obesity-related cancers might be reduced through efforts to prevent and control overweight and obesity. Comprehensive cancer control strategies, including use of evidence-based interventions to promote healthy weight, could help decrease the incidence of these cancers in the United States.

## Introduction

In 2013–2014, approximately one third of adults in the United States were overweight (body mass index [BMI] 25.0–29.9 kg/m^2^) and approximately one third had obesity (BMI ≥30 kg/m^2^) (*1*). Approximately half of U.S. residents are unaware that adults who are overweight or have obesity are at increased risk for cancer (*2*,*3*). The International Agency for Research on Cancer (IARC) states that there is sufficient evidence for an association with excess body fatness, including overweight, obesity, and weight gain, and at least 13 cancers (*3*). These cancers include adenocarcinoma of the esophagus; cancers of the breast (in postmenopausal women), colon and rectum, endometrium (corpus uterus), gallbladder, gastric cardia, kidney (renal cell), liver, ovary, pancreas, and thyroid; meningioma, and multiple myeloma. Overweight and obesity might increase cancer risk through induction of metabolic and endocrine abnormalities, including increases in inflammation and levels of insulin, insulin-like growth factor, and sex hormones (*4*).

Data compiled for the United States Cancer Statistics (USCS) data set (https://nccd.cdc.gov/uscs/) were used to calculate incidence rates in 2014 and trends during 2005–2014 for cancers associated with overweight and obesity (overweight- and obesity-related cancers). In this report, overweight- and obesity-related cancers were defined as those classified by IARC as having sufficient evidence for an association with excess body fatness.

## Methods

The USCS is a compilation of data from multiple sources that is used to report official federal cancer statistics through the USCS web-based report. The USCS data set includes cancer incidence data from CDC’s National Program of Cancer Registries (NPCR) and the National Cancer Institute’s Surveillance, Epidemiology, and End Results (SEER) program. Data on new cancer cases diagnosed during 2005–2014 were obtained from population-based cancer registries affiliated with NPCR and SEER programs in each state and the District of Columbia (DC). Data from DC and all states met USCS publication criteria for 2014, covering 100% of the U.S. population; all states except Nevada met USCS publication criteria each year during 2005–2014, covering approximately 99% of the U.S. population.* Cancer site for cases was classified by anatomic site and histology.^†^ Only cases of invasive cancer were included. Postmenopausal breast cancer was defined as breast cancer diagnosed in women aged ≥50 years.

Population estimates for rate denominators were a modification of annual county population estimates by age, sex, bridged-race, and ethnicity, produced by the U.S. Census Bureau in collaboration with CDC and with support from National Cancer Institute, and aggregated to the state and national levels.^§^ Race bridging is a method used to make multiple-race and single-race data collection systems sufficiently comparable to permit estimation and analysis of race-specific statistics (https://www.cdc.gov/nchs/data/series/sr_02/sr02_135.pdf). Ninety-five percent confidence intervals for rates are presented to allow for informal comparisons among rates, without specifying a referent group. Joinpoint regression (https://surveillance.cancer.gov/joinpoint/), which allowed different slopes for more than one period, was used to calculate changes in rates; trends were quantified by average annual percent change. Because screening for colorectal cancer can reduce colorectal cancer incidence through detection of precancerous polyps before they become cancerous (https://www.uspreventiveservicestaskforce.org/Page/Document/UpdateSummaryFinal/colorectal-cancer-screening2?ds), trends with and without colorectal cancer were analyzed. Rates were estimated by sex, age, race/ethnicity, and U.S. Census region. Trends were age-adjusted and estimated by cancer site, sex, and state. To examine the impact of the change in rates, the number of cases expected during 2006–2014 if rates had remained at 2005 levels was subtracted from the actual number of cases during this period.

## Results

In 2014, approximately 631,604 persons in the United States received a diagnosis of an overweight- or obesity-related cancer (Table 1). This represents 40% of the nearly 1.6 million cancers diagnosed each year (55% of the 799,734 cancers among women and 24% of the 796,752 cancers among males). Overweight- and obesity-related cancer incidence rates were higher among older persons (ages ≥50 years) than younger persons and two thirds of cases occurred among persons aged 50–74 years. The overweight- and obesity-related cancer incidence rate was higher among females (218.1 per 100,000 population) than among males (115.0 per 100,000), partially because endometrial, ovarian, and postmenopausal female breast cancers accounted for 42% (268,091) of overweight-and obesity-related cancers. The rates also varied by race/ethnicity, with higher incidence among non-Hispanic blacks (black) and non-Hispanic whites (white) compared with other groups; however, black males and American Indian/Alaska Native males had higher incidence rates than did white males. Incidence was highest in the Northeast compared with other U.S. Census regions.

**TABLE 1:** Number and annual age-adjusted rate* of overweight- and obesity-related invasive cancer cases,^†^ by selected characteristics — United States,^§^ 2014

Characteristic	Total		Males		Females	
	No.	Rate (95% CI)	No.	Rate (95% CI)	No.	Rate (95% CI)
**Total**	**631,604**	**169.7 (169.3–170.1)**	**194,727**	**115.0 (114.5–115.5)**	**436,877**	**218.1 (217.4–218.7)**
**Age group (yrs)**						
<20	2,230	2.7 (2.6–2.8)	719	1.7 (1.6–1.8)	1,511	3.8 (3.6–4.0)
20–49	60,386	47.2 (46.8–47.6)	20,884	32.5 (32.1–32.9)	39,502	62.1 (61.4–62.7)
50–64	240,299	383.6 (382.0–385.1)	71,518	235.3 (233.6–237.0)	168,781	523.3 (520.8–525.8)
65–74	173,764	658.4 (655.3–661.5)	54,313	440.0 (436.3–443.7)	119,451	850.3 (845.5–855.2)
≥75	154,925	782.1 (778.2–786.0)	47,293	592.1 (586.8–597.5)	107,632	910.4 (904.9–915.8)
**Race/Ethnicity^¶^**						
White	470,789	170.9 (170.4–171.4)	144,456	114.2 (113.6–114.8)	326,333	222.3 (221.5–223.1)
Black	71,847	186.5 (185.1–188.0)	22,129	134.2 (132.3–136.1)	49,718	226.3 (224.3–228.4)
American Indian/Alaska Native	3,970	162.5 (157.2–167.9)	1,376	121.9 (115.1–129.0)	2,594	197.3 (189.5–205.3)
Asian/Pacific Islander	23,193	128.4 (126.7–130.1)	6,904	87.7 (85.6–89.9)	16,289	162.2 (159.7–164.8)
Hispanic	55,778	150.6 (149.3–152.0)	17,990	108.8 (107.1–110.6)	37,788	188.0 (186.0–189.9)
**Census region^††^**						
Northeast	127,436	185.3 (184.2–186.3)	37,739	122.8 (121.5–124.0)	89,697	239.2 (237.6–240.9)
Midwest	140,687	173.8 (172.9–174.8)	43,173	117.6 (116.5–118.7)	97,514	224.1 (222.7–225.6)
South	230,431	165.7 (165.0–166.4)	73,138	115.5 (114.7–116.4)	157,293	209.5 (208.4–210.5)
West	133,050	159.7 (158.8–160.6)	40,677	105.5 (104.5–106.6)	92,373	208.9 (207.5–210.3)

**Abbreviation:** CI = confidence interval.

* Per 100,000 persons, age-adjusted to the 2000 U.S. standard population.

^†^ Overweight- and obesity-related cancers include adenocarcinoma of the esophagus; cancers of the breast [in postmenopausal women], colon and rectum, endometrium, gallbladder, gastric cardia, kidney, liver, ovary, pancreas, and thyroid; meningioma; and multiple myeloma.

^§^ Cancer incidence compiled from cancer registries that meet the data quality criteria for all invasive cancer sites combined (covering 100% of the U.S. population).

^¶^ Mutually exclusive racial/ethnic groups are based on information about race/ethnicity that was collected separately and combined for this report. The white, black, American Indian/Alaska Native, and Asian/Pacific Islander race categories are all non-Hispanic. Hispanic persons can be any race. Rates are not presented for those with unknown or other race or unknown ethnicity.

^††^ *Northeast*: Connecticut, Maine, Massachusetts, New Hampshire, New Jersey, New York, Pennsylvania, Rhode Island, and Vermont. *Midwest*: Illinois, Indiana, Iowa, Kansas, Michigan, Minnesota, Missouri, Nebraska, North Dakota, Ohio, South Dakota, and Wisconsin. *South*: Alabama, Arkansas, Delaware, District of Columbia, Florida, Georgia, Kentucky, Louisiana, Maryland, Mississippi, North Carolina, Oklahoma, South Carolina, Tennessee, Texas, Virginia, and West Virginia. *West*: Alaska, Arizona, California, Colorado, Hawaii, Idaho, Montana, Nevada, New Mexico, Oregon, Utah, Washington, and Wyoming.

Among cancers affecting both males and females, incidence rates in 2014 were higher among males than among females for colorectal cancer (44.1 per 100,000 versus 33.7 per 100,000), kidney cancer (20.9 versus 10.6), pancreatic cancer (14.4 versus 11.1), liver cancer (11.2 versus 3.4), adenocarcinoma of the esophagus (5.4 versus 0.8), multiple myeloma (7.5 versus 4.9), and gastric cardia cancer (3.6 versus 0.8) (Table 2). Females had higher rates than did males of thyroid cancer (21.3 versus 7.4) and gallbladder cancer (1.4 versus 0.8). Among the three overweight- and obesity-related cancers that affect females only, incidence rates were higher for postmenopausal breast cancer (92.6 per 100,000) than for endometrial cancer (26.5 per 100,000) and ovarian cancer (11.0 per 100,000). By site, incidence rates decreased significantly each year for meningioma (-3.8% per year), colorectal cancer (-2.9%), and ovarian cancer (-2.0%). Incidence rates increased significantly each year during this period for six cancers: thyroid cancer (4.0% per year), liver cancer (2.9%), gastric cardia cancer (1.2%), endometrial cancer (1.1%), pancreatic cancer (0.8%), and kidney cancer (0.7%). The incidence rates were stable for adenocarcinoma of the esophagus, gallbladder cancer, multiple myeloma, and postmenopausal breast cancer. The increase in risk for cancer per 1 kg/m^2^ increase in BMI ranged from 1% each for thyroid and ovarian cancers to 9% for adenocarcinoma of the esophagus.

**TABLE 2:** Age-adjusted incidence of overweight- and obesity-related invasive cancer, changes in rates, and estimated percent increase in cancer risk associated with change in BMI, by cancer site and sex — United States,* 2005 and 2014

Cancer site	%	2005	2014	2005–2014		% Increase in risk for cancer per 1 kg/m^2^ increase in BMI^¶^
		Rate^†^ (95% CI)	Rate^†^ (95% CI)	% Change in rates	Average annual percent change in rates^§^	
**Breast [in postmenopausal women]**	31	90.5 (90.1–91.0)	92.6 (92.2–93.0)	2	0.2	2
**Colon and rectum**	22	49.7 (49.4–49.9)	38.4 (38.2–38.6)	-23	-2.9^§^	2
Male		58.1 (57.7–58.5)	44.1 (43.7–44.4)	-24	-3.1^§^	
Female		43.1 (42.8–43.4)	33.7 (33.4–34.0)	-22	-2.8^§^	
**Kidney (renal cell)**	9	14.4 (14.2–14.5)	15.4 (15.2–15.5)	7	0.7^§^	5
Male		19.5 (19.3–19.7)	20.9 (20.7–21.1)	7	0.7^§^	
Female		10.2 (10.0–10.3)	10.6 (10.4–10.7)	4	0.4	
**Endometrium (corpus uterus) (female only)**	8	23.9 (23.7–24.1)	26.5 (26.3–26.8)	11	1.1^§^	8
**Thyroid**	8	10.3 (10.2–10.4)	14.4 (14.3–14.6)	40	4.0^§^	1
Male		5.3 (5.1–5.4)	7.4 (7.2–7.5)	40	4.0^§^	
Female		15.2 (15.0–15.4)	21.3 (21.1–21.5)	40	4.0^§^	
**Pancreas**	7	11.7 (11.6–11.9)	12.6 (12.5–12.7)	7	0.8^§^	2
Male		13.3 (13.1–13.5)	14.4 (14.2–14.5)	8	0.8^§^	
Female		10.5 (10.3–10.6)	11.1 (10.9–11.2)	6	0.7^§^	
**Multiple myeloma**	4	5.6 (5.5–5.7)	6.0 (6.0–6.1)	8	1.1	2
Male		6.9 (6.7–7.0)	7.5 (7.3–7.6)	9	1.2^§^	
Female		4.6 (4.5–4.8)	4.9 (4.8–5.0)	6	1.1^§^	
**Liver**	4	5.5 (5.4–5.6)	7.0 (7.0–7.1)	29	2.9^§^	5
Male		8.8 (8.6–8.9)	11.2 (11.0–11.3)	28	2.9^§^	
Female		2.7 (2.6–2.8)	3.4 (3.3–3.5)	26	2.5^§^	
**Ovary (female only)**	3	13.1 (12.9–13.2)	11.0 (10.8–11.2)	-16	-2.0^§^	1
**Adenocarcinoma of the esophagus**	2	2.9 (2.8–2.9)	2.9 (2.8–2.9)	-1	-0.5	9
Male		5.5 (5.4–5.7)	5.4 (5.2–5.5)	-3	-0.7^§^	
Female		0.8 (0.7–0.8)	0.8 (0.7–0.8)	2	-0.4	
**Gastric cardia**	1	1.9 (1.9–2.0)	2.1 (2.0–2.1)	8	1.2^§^	4
Male		3.4 (3.3–3.5)	3.6 (3.5–3.7)	7	1.1^§^	
Female		0.8 (0.7–0.8)	0.8 (0.8–0.9)	6	0.8^§^	
**Gallbladder**	1	1.1 (1.1–1.2)	1.1 (1.1–1.2)	-1	-0.1	5
Male		0.8 (0.7–0.8)	0.8 (0.8–0.8)	3	0.1	
Female		1.4 (1.4–1.5)	1.4 (1.3–1.5)	-1	-0.1	
**Meningioma**	<1	0.1 (0.1–0.2)	0.1 (0.1–0.1)	-29	-3.8^§^	4
Male		0.1 (0.1–0.1)	0.1 (0.1–0.1)	-17	-2.7^§^	
Female		0.2 (0.1–0.2)	0.1 (0.1–0.1)	-35	-4.0^§^	
**All overweight- and obesity-related cancers**	—	173 (173–174)	170 (169–170)	-2	-0.3^§^	—
**All overweight- and obesity-related cancers except colorectal cancer**	—	123 (123–124)	132 (131–132)	7	0.8^§^	—
**Cancers not related to overweight and obesity**	—	306 (305–306)	267 (267–268)	-13	-1.4^§^	—

**Abbreviations**: BMI = body mass index calculated as weight in kilograms/height in meters squared (kg/m^2^); CI = confidence interval.

* Cancer incidence compiled from cancer registries that meet the data quality criteria for all invasive cancer sites combined for all years during the period 2005 to 2014 (covering 99% of the U.S. population).

^†^ Per 100,000 persons, age-adjusted to the 2000 U.S. standard population.

^§^ Significant at p<0.05. Trends were measured with average annual percent change in rates and were considered to increase or decrease if p<0.05; otherwise trends were considered stable.

^¶^ Based on relative risk estimates from pooled epidemiologic studies (reviewed in http://www.nejm.org/doi/full/10.1056/NEJMsr1606602) and in the World Cancer Research Fund Continuous Update Project (http://www.wcrf.org/int/research-we-fund/continuous-update-project-cup).

**FIGURE 1:**
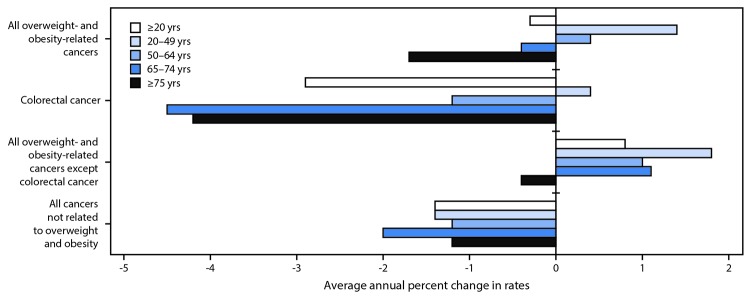
Average annual percent change* in overweight- and obesity-related invasive cancer incidence rates^†^ among adults — United States,^§^ 2005–2014 * Average annual percent change (AAPC) was calculated using joinpoint regression, which allowed different slopes for two periods; the year at which slopes changed could vary by age. All AAPCs were significantly different from zero (p<0.05) except for colorectal cancer in persons aged 20–49 years. ^†^ Overweight- and obesity-related cancer (adenocarcinoma of the esophagus; cancers of the breast [in postmenopausal women], colon and rectum, endometrium, gallbladder, gastric cardia, kidney, liver, ovary, pancreas, and thyroid; meningioma; and multiple myeloma) rates were calculated with and without colorectal cancer because colorectal cancer screening can detect precancerous polyps before they become cancerous, which might affect cancer incidence. ^§^ Cancer incidence compiled from cancer registries that meet the data quality criteria for all invasive cancer sites combined for each year during the period 2005–2014 (covering 99% of the U.S. population).

**FIGURE 2:**
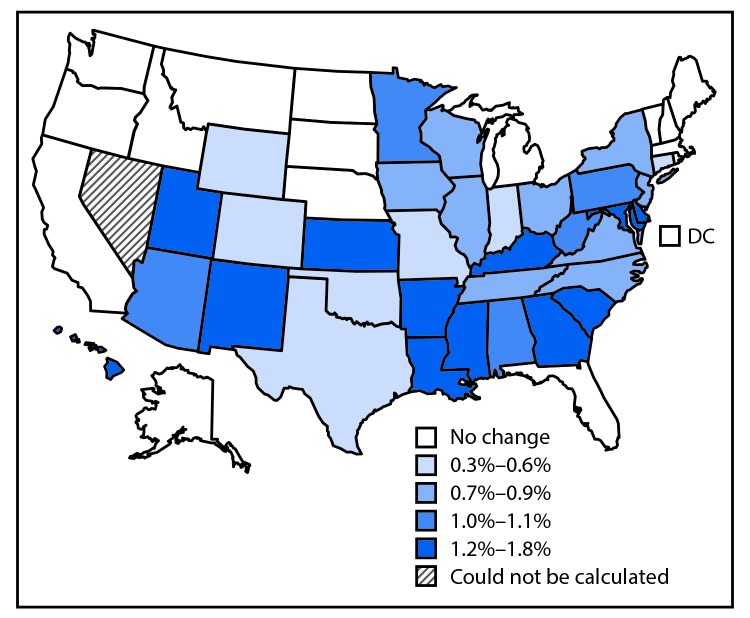
Average annual percent change in incidence of overweight- and obesity-related cancers,* by quartile — United States, 2005–2014 * Except colorectal cancer.

During 2005–2014, declines were observed in the overall incidence of overweight- and obesity-related cancers (-2%), colorectal cancer (-23%), and cancers not known to be related to overweight and obesity (-13%) (Table 2). Increased use of colorectal cancer screening tests likely contributed to the decline in colorectal cancer; when colorectal cancer was excluded from overweight- and obesity-related cancers, a 7% increase in overall incidence was observed. The trends varied substantially by age group: the rate for all overweight- and obesity-related cancers increased significantly among persons aged 20–49 years and 50–64 years, and decreased among those aged 65–74 years and ≥75 years; colorectal cancer rates declined in all age groups except in persons aged 20–49 years; and rates for overweight- and obesity-related cancers (excluding colorectal cancers) increased among all age groups except persons aged ≥75 years (Figure 1). Because of reductions in colorectal cancer rates, approximately 224,800 cases have been averted since 2005. However, during this same period, 211,800 excess cases from other overweight- and obesity-related cancers have occurred. Incidence rates of overweight- and obesity-related cancers (excluding colorectal cancer) increased significantly in 32 states (0.3%–1.8%), and did not change in 16 states and the District of Columbia (Figure 2).

## Conclusions and Comments

Overweight- and obesity-related cancers accounted for 40% of all cancers diagnosed in 2014, and varied substantially across demographic groups. Endometrial, ovarian, and postmenopausal female breast cancers accounted for 42% of new cases of overweight-and obesity-related cancers in 2014, which is reflected in the higher overall incidence of overweight- and obesity-related cancers among females. For cancers that occurred among both males and females, however, the incidence of most cancers was higher in males.

Meningioma declined the most (-3.8% per year) during 2005–2014; however, this cancer accounted for <1% of overweight- and obesity-related cancers among males and females. The second largest decline was in the rate of colorectal cancer, which accounted for approximately 22% of overweight- and obesity-related cancers; this trend likely influenced the overall decline in the incidence of overweight- and obesity-related cancers during 2005–2014. National data have demonstrated an increase in colorectal cancer screening (*5*), which might have contributed to the decline in colorectal cancer incidence through detection of precancerous polyps, which can then be removed before becoming cancerous. When colorectal cancer was excluded from the trend analysis, overweight- and obesity-related cancer incidence increased among all age groups except persons aged ≥75 years. The increase in obesity-related cancer incidence coincides with an increase in the prevalence of obesity since 1960 in the United States with larger absolute percentage increases from 1960 to 2004 than from 2005 to 2014 (*1*). The prevalence of overweight during this later period remained stable. These historical and current trends in overweight and obesity and cancers related to excess weight reflect the continued need for public health strategies to prevent and control overweight and obesity in children and adults and help communities make it easier for people to be physically active and eat healthfully.

There is consistent evidence that a high BMI is associated with cancer risk. Persons who are overweight or have obesity are nearly twice as likely as are healthy-weight (BMI = 18.5–24.9kg/m^2^) persons to develop adenocarcinoma of the esophagus and cancers of the gastric cardia, liver, and kidney (*6*–*9*). Persons who have obesity are approximately 30% more likely to develop colorectal cancer than are persons with healthy weight (*10*). Women who are overweight or have obesity are approximately two to four times as likely as are women with healthy weight to develop endometrial cancer (*11*).

Observational studies have provided evidence that even a 5-kg (11 pound) increase in weight since early adulthood is associated with increased risk for overweight- and obesity-related cancers (*12*). Maintaining a healthy weight throughout life has been associated with a reduction in risk of these cancers (*3*). However, the population effect of weight loss interventions on cancer risk might not be observable for at least a decade (*4*). In studies evaluating the effect of weight change on risks for endometrial cancer and breast cancer after long-term follow-up, weight loss was associated with reduced risks for both types of cancer among postmenopausal women (*13*,*14*).

Without intensified nationwide efforts to prevent and treat overweight and obesity, the high prevalence of excess weight might impede further declines in overall cancer incidence (*15*). These efforts include investing in addressing both social and behavioral determinants of health, such as unemployment and disparities in education and housing, to achieve better population health (https://nam.edu/addressing-social-determinants-of-health-and-health-disparities-a-vital-direction-for-health-and-health-care/). Eating a healthy diet and engaging in sufficient physical activity are important components of behavioral strategies to maintain a healthy weight. Population-based strategies to prevent and reduce overweight and obesity include helping persons of all ages meet dietary (https://health.gov/dietaryguidelines/2015/guidelines) and physical activity (https://health.gov/PAGuidelines) guidelines by supporting healthy eating and active living in a variety of settings, including communities, worksites, schools, and early care and education facilities. Strategies to provide support for these settings have been recommended by a number of public health entities including CDC (https://www.cdc.gov/mmwr/preview/mmwrhtml/rr5807a1.htm), the National Academy of Medicine (*16*), and the Community Preventive Services Task Force (https://www.thecommunityguide.org/topic/obesity). Health care providers could encourage patients to maintain healthy weights throughout their lifespans. To help treat obesity, the U.S. Preventive Services Task Force recommends that clinicians screen all adults for obesity and either offer patients who have obesity intensive, multicomponent behavioral interventions or refer them to programs that offer these services (https://www.uspreventiveservicestaskforce.org/Page/Document/UpdateSummaryFinal/obesity-in-adults-screening-and-management); similar recommendations exist for children aged ≥6 years (https://www.uspreventiveservicestaskforce.org/Page/Document/UpdateSummaryFinal/obesity-in-children-and-adolescents-screening).

The CDC’s National Comprehensive Cancer Control Program supports comprehensive cancer control efforts in all 50 states, DC, eight tribes and tribal organizations, and seven U.S. territories and Pacific Island jurisdictions; these efforts include policy, systems, and environmental changes that promote physical activity and healthy food options in communities. A review of cancer control plans implemented by grantees revealed that 89% include goals or strategies related to nutrition or physical activity to reduce cancer risk, with 82% including both (*17*). Other CDC programs, such as the State Public Health Action’s Program, address diet, physical activity, and obesity more broadly (https://www.cdc.gov/nccdphp/dnpao/state-local-programs/state-public-health-action.html). Maintaining and strengthening these programmatic activities might help reduce the burden of overweight- and obesity-related cancer.

The findings in this report are subject to at least five limitations. First, the weights and BMI histories of cancer patients were not known. Second, because race and ethnicity data are abstracted from medical records, they are subject to misclassification (https://www.cdc.gov/cancer/npcr/uscs/technical_notes/interpreting/race.htm). Third, whereas IARC’s most recent report was used to define overweight- and obesity-related cancer, this might underestimate the actual burden, because evidence is still accumulating related to the association of overweight and obesity with other cancers (*3*). Fourth, many different risk factors might contribute to development of overweight- and obesity-related cancers, such as genetic mutations; chronic infections; and tobacco, hormone, and alcohol use (*2*). Changes in these other risks, as well as in cancer screening rates, might have affected the number of cancer cases and the trends described in this report. Finally, although this report tracks overweight- and obesity-related cancers, it does not estimate what proportion of these cancers are attributable to overweight and obesity.

The incidence of overweight- and obesity-related cancers (excluding colorectal cancer) increased significantly among persons aged 20–74 years during 2005–2014, mirroring increases of obesity observed since 1960 (*1*). Multilevel approaches to comprehensive cancer control that address social determinants of health and include evidence-based interventions that address healthy weight and other cancer risk factors might help reduce the burden of cancer and other chronic diseases in the United States.

**Key Points**

• Overweight and obesity are associated with increased risk of at least 13 different types of cancer.

• Overweight- and obesity-related cancers accounted for 40% of all cancers diagnosed in 2014.

• About 55% of cancers diagnosed in women and 24% of those diagnosed in men are overweight- and obesity-related cancers.

• The incidence of overweight- and obesity-related cancers (excluding colorectal cancer) increased significantly among persons aged 20–74 years during 2005–2014.

• The findings emphasize the importance of intensifying nationwide efforts to prevent and treat overweight and obesity.

• Multilevel approaches to comprehensive cancer control that address social determinants of health and include evidence-based interventions that address healthy weight and other cancer risk factors might help reduce the burden of cancer and other chronic diseases.

## Footnotes

* Cancer registries demonstrated that cancer incidence data were of high quality by meeting five United States Cancer Statistics publication criteria: 1) ≤5% of cases ascertained solely on the basis of death certificate; 2) ≤3% of cases missing information on sex; 3) ≤3% of cases missing information on age; 4) ≤5% of cases missing information on race; and 5) ≥97% of registry’s records passed a set of single-field and inter-field computerized edits that test the validity and logic of data components (https://www.cdc.gov/cancer/npcr/uscs/technical_notes/criteria.htm).

† Cases were first classified by anatomic site using the *International Classification of Diseases for Oncology, Third Edition* (http://codes.iarc.fr/) then cases with hematopoietic histologies were classified using the *World Health Organization Classification of Tumours of Haematopoietic and Lymphoid Tissues, Fourth Edition* (http://www.bloodjournal.org/content/117/19/5019?sso-checked=true#T1).

§ Population estimates incorporate bridged single-race estimates derived from the original multiple race categories in the 2010 U.S. Census (https://seer.cancer.gov/popdata/index.html).
